# The Metropolitan Hospital

**Published:** 1920-11-20

**Authors:** 


### The Metropolitan Hospital.

The Lord Mayor and Lady Mayoress lately entertained at the Mansion House a group of forty active friends of the Metropolitan Hospital, when Mr. Sydney A. Young, Chairman of the Appeal Committee, presented the Lady Mayoress with an illuminated address for her help to the Appeal Committee. Thanking her guests, who, she said, deserved the praise, the Lady Mayoress announced that a dance, for the benefit of the hospital, would be held in a Livery Hall early in December.

